# Potentiation of motor sub-networks for motor control but not working memory: Interaction of dACC and SMA revealed by resting-state directed functional connectivity

**DOI:** 10.1371/journal.pone.0172531

**Published:** 2017-03-09

**Authors:** Vaibhav A. Diwadkar, Avisa Asemi, Ashley Burgess, Asadur Chowdury, Steven L. Bressler

**Affiliations:** 1 Dept. of Psychiatry & Behavioral Neurosciences, Wayne State University School of Medicine, Detroit, Michigan, United States of America; 2 Center for Complex Systems and Brain Sciences, Florida Atlantic University, Boca Raton, Florida, United States of America; 3 Department of Psychology, Florida Atlantic University, Boca Raton, Florida, United States of America; National University of Defense Technology College of Mechatronic Engineering and Automation, CHINA

## Abstract

The dorsal Anterior Cingulate Cortex (dACC) and the Supplementary Motor Area (SMA) are known to interact during motor coordination behavior. We previously discovered that the directional influences underlying this interaction in a visuo-motor coordination task are asymmetric, with the dACC→SMA influence being significantly greater than that in the reverse direction. To assess the specificity of this effect, here we undertook an analysis of the interaction between dACC and SMA in two distinct contexts. In addition to the motor coordination task, we also assessed these effects during a (n-back) working memory task. We applied directed functional connectivity analysis to these two task paradigms, and also to the rest condition of each paradigm, in which rest blocks were interspersed with task blocks. We report here that the previously known asymmetric interaction between dACC and SMA, with dACC→SMA dominating, was significantly larger in the motor coordination task than the memory task. Moreover the asymmetry between dACC and SMA was reversed during the rest condition of the motor coordination task, but not of the working memory task. In sum, the dACC→SMA influence was significantly greater in the motor task than the memory task condition, and the SMA→dACC influence was significantly greater in the motor rest than the memory rest condition. We interpret these results as suggesting that the potentiation of motor sub-networks during the motor rest condition supports the motor control of SMA by dACC during the active motor task condition.

## Introduction

How are brain networks potentiated for action? As with the muscles in the body, the potential for dynamics in the brain may be encoded in the relationship between the system’s rest state and its active state. This relationship has been extensively discussed in terms of the metabolic demands of the brain in both rest and task-active states, particularly from the perspective of the fMRI signal [1]. The explosion of interest in resting-state fMRI signals can in part be traced to these initial theoretical discussions. Nevertheless, much of resting-state fMRI (rsfMRI) research has been driven by the search for understanding default mode function in the brain [2], or in discovering network structure from spontaneous fluctuations in the fMRI signal [3, 4]. In large part, these initiatives have uncovered general structural constraints driving rsfMRI fluctuations that are spontaneous, induced by physiological stimulation [5], or constrained by task-active processing [6]. Yet, a parallel literature continues to investigate resting-state connectivity and its relationship to network function in the task-active state [7, 8]. These investigations indicate that functional connectivity between networks in the rest state, is predictive of the same in the task state [9].

The current investigation is motivated by previous work on rsfMRI and task-based fMRI, and employs directed functional connectivity (dFC) analysis [10] to assess network interactions between constituents of the brain’s motor system during task and rest. We define dFC as the directed influence from one network constituent to another in the brain as derived from previously established quantitative models [11]. The analysis of dFC always involves computing directed influence in both directions between the constituents of a pair, and may potentially reveal asymmetric interactions between them. In the present study, dFC in the human motor cortex was perturbed using a simple uni-manual visuomotor paradigm that has been previously shown to induce asymmetric dFC from the dorsal anterior cingulate cortex (dACC) to the supplementary motor area (SMA) [12]. We refer to this dACC → SMA functional connection as a “descending” functional pathway. This functional pathway may follow anatomical pathways, given dACC’s position in the hierarchy of the motor system [13].

Here we show that a task-induced top-down dominant dFC asymmetry in this descending pathway is complimented by a bottom-up dominant dFC asymmetry in the ascending (SMA → dACC) pathway during rest epochs that “bookend” task epochs. We also demonstrate that this effect is relatively specific to motor paradigms, and does not generalize to working memory paradigms where motor demands are secondary to the fundamental demands of working memory [14, 15]. We interpret our results to suggest that brain network interactions at rest may induce directional potentiation of complimentary task-related processing. We advocate for the use of more complex functional assessments of brain sub-networks, in both the task-active and rest states, to unravel how task-specific resting-state brain network interactions might potentiate brain activity during task-active states and inform our understanding of the emergence of cognitive architectures.

## Materials and methods

### Participants

The research meets all applicable standards for the ethics of experimentation and research integrity and was conducted in concordance with established guidelines at the native institutions. Ten subjects provided written informed consent or assent to participate in the fMRI studies. Consent or assent was recorded on forms that were approved by the Human Investigative Committee at the Wayne State University School of Medicine. Participants (Mean Age: 14.1 yrs; Range 8.4–18.6 yrs) were drawn from the metro Detroit area and were monetarily compensated for their participation. The subject sample was assembled as part of the healthy cohort for a study assessing development effects in clinical syndromes. None of the participants had psychiatric or neurological diagnoses, being screened with the Schedule for Affective Disorders and Schizophrenia for School-Age Children-Present and Lifetime Version (K-SADS-PL) (Kaufman et al. 1997). All were predominantly right handed as evaluated using the structured Neurological Evaluation Scale (Buchanan & Heinrichs 1989). Even though developmental questions were not central to our motivations (which were squarely focused on network architectures), our analyses did address potential developmental trends. We also discuss how the subjects’ characteristics may constraint the external validity of our study.

### Image acquisition

Both fMRI and structural MRI data were collected during the course of the study (4T Bruker MedSpec; TR: 2s, TE: 30 ms., matrix: 64 x 64, 24 slices, 1 mm gap, voxels: 3.8 x 3.8 x 4.0 mm; 3D T_1_-weighted MPRAGE sequence (TR = 2200 ms, TE = 2.56 ms, flip angle = 13°, FOV = 208×256 mm, voxel size = 1×1×1 mm). Head motion was minimized using foam inserts surrounding each participant’s head. Participants wore earplugs to reduce scanner noise. During each scan, experimental paradigm stimuli were projected onto a screen mounted in the scanner using Presentation (Neurobehavioral Systems, Inc.). For the motor task, responses were acquired on the receptive surface (extent: 33 x 33 mm) of a fiber-optic response touchpad (Current Design Systems, Inc.). For the working memory task, subjects signaled their responses by button press on a response box.

### Tasks of interest

#### Motor control

Subjects tapped the forefinger of their right hand in response to a flashing white stimulus presented at the center of the display (RGB:255,255,255; extent: 34 x 32 mm; subtended visual angle: ~17°; stimulus duration: 100 ms). Four behavioral conditions were employed through a factorial combination of the frequency of the presented stimulus (1 Hz or 0.5 Hz) and the periodicity of its presentation (Periodic or Pseudo-random intervals between stimuli). Inter-stimulus intervals (in s) for the Pseudo-random epochs (either 1 Hz or 0.5 Hz) were created by pseudo-randomly sampling values from Gaussian distributions (μ = 1.0 sec and σ = 0.5 sec, or μ = 2.0 sec and σ = 1.0 sec). Stimulus Onset Asynchronies (SOAs) during Pseudo-random epochs were adjusted so that the average frequency of the elicited response (and therefore the number of elicited responses) was equal to the periodic counterpart. Interspersed between task epochs were extended (30 s, 2 total) resting epochs (which were also conditions of interest in our study). During the resting epochs, subjects were instructed to fixate on a cross hair in the center of the field of vision. The task alternated between the eight motor epochs (30s duration each, 2 per condition, thus contributing 120 images to the analysis) and the long rest epochs, with brief interludes (10 s) for rest. The total task duration was 6 minutes and 50 s with all data collected over a single run.

#### Working memory

An established visual n-back paradigm was used to assess working memory, during which letter stimuli were projected in sequence (Presentation Time: 500 ms; ISI: 2500 ms) and subjects signaled by button press if the letter was a target letter (0-Back condition) or the same as the one shown two letters previously in the sequence (2-Back condition). A block design was employed wherein conditions were blocked (30 s/epochs) for each of the 0- and 2-Back conditions (5 epochs each, 10 epochs total, thus contributing 150 images to the analysis), and, as with the motor task, extended resting epochs of interest were included (20 s; 10 epochs total), during which subjects were instructed to fixate on a cross hair in the center of the field of vision. The total task duration was 8 minutes and 20 s with all data collected over a single run.

### fMRI data processing

MR images were preprocessed and analyzed with SPM 8 (Statistical Parametric Mapping, Wellcome Department of Imaging and Neuroscience, London, UK) using established methods of spatial and temporal preprocessing including orientation to the anterior-posterior commissure axis, realignment, slice timing correction, and normalization. Using the T_1_ weighted image as the structural reference, EPI images were manually oriented to the AC-PC line with the reorientation vector applied across the EPI image set. Following slice timing correction, images were realigned to a mean reference image to correct for head movement. Then, the high-resolution T_1_ weighted image was normalized to the MNI template, with the resultant deformations subsequently applied to the EPI images for normalization. Low frequency components were removed using a high-pass filter (128 s) and images were spatially smoothed using a Gaussian filter (8 mm full-width half maximum; FWHM). An autoregressive AR(1) model was used to account for serial correlation, and regressors modeled as box-car vectors (for each of the task-related blocks for each task) were convolved with a canonical hemodynamic reference wave form. Subjects’ head motion was within accepted limits (< 4 mm) assessed for each participant and acquisition (motor and memory). Furthermore, in all first level models, the effects of motion were modeled by including the six motion parameters as covariates of no interest.

#### Activation analyses

For each of the tasks, first-level activation maps were submitted to second-level random effects analyses isolating activations during each of the Motor and Memory paradigms, within each of the dACC, SMA and left M1 [16]. Cluster-level thresholding (*p* < .05, cluster level; *p* < .01, cluster forming threshold) was applied to identify significant clusters [17]. All analyses were spatially constrained respecting the relative homogeneity of function within these *a priori* regions of interest. Significant clusters within each region were identified using AlphaSim [17], by estimating the minimum cluster extent in order for activated clusters to be rejected as false positive (noise-only) clusters. This chosen approach performs a Monte Carlo alpha probability simulation, computing the probability of a random field of noise (after taking into account the spatial correlations of voxels based on the image smoothness within each region of interest estimated directly from the data set) to produce a cluster of a given size, after the noise is thresholded at a given level. Thus, instead of using the individual voxel probability threshold alone in achieving the desired overall significance level, the method uses a combination of both probability thresholding and minimum cluster size thresholding. The underlying principle is that true regions of activation will tend to occur over contiguous voxels within a region of relative functional homogeneity, whereas noise has much less of a tendency to form clusters of activated voxels. Using the mass analyses of resting state data, it has recently been suggested that cluster based methods result in inflated false-positive rates identifying clusters that are spurious [18]. However, our goal in conducting activation analyses was not to discover new regions engaged in a task: rather these analyses were deployed to depict task-related engagement in our regions of interest, and as such were a prelude to subsequently described time series analyses.

#### Time series analyses

To maximize sensitivity for accommodating inter-subject variations in peak activation loci, time series were extracted from first-level activation profiles for each subject (*p*_FWE_ < .05). From the first-level activation maps, within each of the dACC, SMA and M1, effects of interest contrasts were used to extract eigenvariate time series based on the significance of the *F* statistic from the comparison of all task blocks (S1 & S2 Files). For each task, the time series represented the weighted means of modeled effects, and is robust under the heterogeneity of the task blocks. Directional functional connectivity (dFC) was estimated from MultiVariate AutoRegressive (MVAR) models. Separate MVAR models were estimated from eigenvariate time series of the ROIs for each of the Rest blocks in each participant. The MVAR model order was one for every model [19]. Higher model orders were not tested because model order one was sufficient for meeting the aims of the study, and the interpretation of between-condition results from higher models would be unlikely to capture network interactions at time scales that are proximate to the cognitive neurodynamics (that are observed at shorter time scales, typically in the millisecond range. The strength of dFC between ROIs, either during a task paradigm or its corresponding rest condition, was estimated by the magnitude of a *t*-statistic derived by significance testing of the corresponding MVAR model coefficient (*glm* function, R software). This strength served as a metric for the dynamic causal relationship between the time series, similar to Granger Causality derived from the MVAR model, and was separately computed for each of the Rest and the Task (Motor and Working Memory) conditions. A sliding window approach to MVAR modeling was adapted within each condition: first, the task blocks from each condition were concatenated; then, separate MVAR models were estimated for each pair of time points, avoiding block boundaries; finally, the dFC values from each time point pair were averaged to yield the final dFC value.

The analysis framework was structured to identify all dFC influences among dACC, SMA, and M1 in order to delineate task-related and resting subnetworks. The following analyses were performed:

1. Subnetworks (dACC, SMA, M1) in the Rest states of the Task paradigms (Motor vs. Memory) were compared, allowing us to investigate whether subnetworks were differentially modulated by task context at rest.
2. Subnetworks (dACC, SMA, M1) in the Task states (Motor vs. Memory) were compared, allowing us to investigate whether subnetworks were differentially modulated by the tasks.
3. Subnetworks (dACC, SMA, M1) were compared between conditions within each Task paradigm (Motor: Periodic vs. Random; Memory: 2-Back vs. 0-Back), allowing us to assess between-condition, within-task modulation of subnetworks. This type of comparison included that between the Rest and Task states, allowing us to investigate the relation of resting-state and task-related subnetworks.

All of the above analyses were structured as two-way repeated measures analyses of variance, with Direction (e.g., dACC → SMA vs. SMA → dACC) and Condition (e.g., Motor Rest vs. Memory Rest) as repeated factors. Significant interactions were unpacked using post-hoc *t*-tests, particularly to assess conditional effects within each direction. Finally, we also assessed age effects on dFC, to investigate whether age modulated changes in estimated dFC. Statistical control within each family of ANOVAs was maintained using Bonferroni correction to account for multiple comparisons across tests within the family, and any post-hoc *t*-tests that were subsequently conducted.

## Results

### Behavioral effects

#### Motor control

Response latencies to the probe were analyzed in a two-way repeated measures analysis of co-variance, with Periodicity (Periodic vs. Pseudo-random) and Frequency (.5 vs. 1. Hz) as within-subjects factors, and age as covariate. This analysis revealed a significant effect of Periodicity (*F*_1,8_ = 25.6, *p* < .001, MSe = 2144.12) with a large effect size (partial η^2^ = .76). These results were driven by a significant increase in latencies associated with the pseudorandom relative to the periodic condition (356 ms. vs. 275 ms.). Effects relating to Frequency, Age and interactions between each of the Factors and age were not significant (*p*s < .05).

#### Working memory

Sensitivity on the working memory task was assessed using the sensitivity index *d*’ [20] to evaluate participants ability to discriminate targets from distracters during the memory paradigm. Analysis revealed that sensitivity was significantly greater than zero (*t*_9_ = 15.96, *p* < .001). Additional regression analyses suggested that sensitivity increased with age (*F*_1,8_ = 6.77, *p* < .05).

### fMRI activation maps

**Fig 1:**
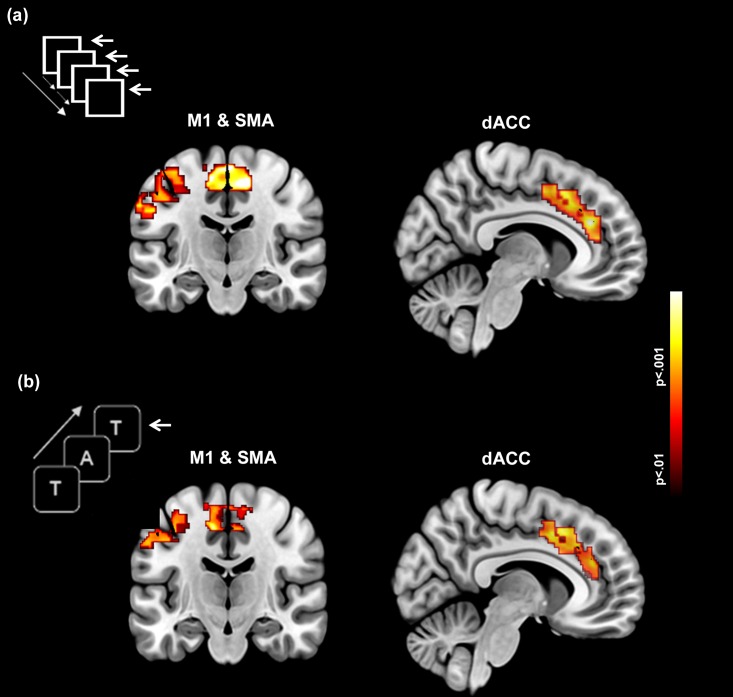
Activation maps are depicted in dACC, SMA and M1 for each of the (a) Motor and (b) Memory paradigms. Each task is schematically depicted in the accompanying graphic. In (a) subjects made a motor response when the visual probe flashed (arrow denotes the finger response). In (b), the motor control demands were secondary to the memory component. Subjects made a motor response when the current memoranda matched the one presented 2 items previously in the sequence (2-Back). As seen across panels, both tasks resulted in robust activation in each of the three regions of interest.

To depict group-level activations, first-level activation maps across conditions, were submitted to second-level random effects analyses isolating activations during each of the Motor and Memory paradigms, within each of the dACC, SMA and left M1 [16]. Cluster-level thresholding (*p* < .05, cluster level; *p* < .01, cluster forming threshold) was applied to identify significant clusters [17]. The second-level group activation maps for each of the Motor and Memory paradigms are depicted in Fig 1, showing significant clusters of activation in the dACC (sagittal depiction), and M1 and the SMA (coronal depiction)[16]. Statistical and locational information are provided in Table 1.

**Table 1:** Statistical and location information are provided for the activation peaks and clusters depicted in Fig 1 for the a) Motor and b) Memory tasks.

Region of interest	MNI coordinates (x, y, z)			*t* score	Cluster extent	*p* (peak)
***Motor*** (Fig 1a)						
dACC	8	34	24	7.45	2465	0.000
SMA	8	-22	52	9.57	3240	0.000
LM1	-64	-12	30	11.51	1273	0.000
***Memory*** (Fig 1b)						
dACC	3	23	43	8.25	2992	0.000
SMA	2	21	43	8.27	4297	0.000
LM1	-28	-22	48	8.14	1455	0.000

### Connectivity analyses (dACC ←→ SMA)

#### Resting-state dFC of dACC ←→ SMA

**Fig 2:**
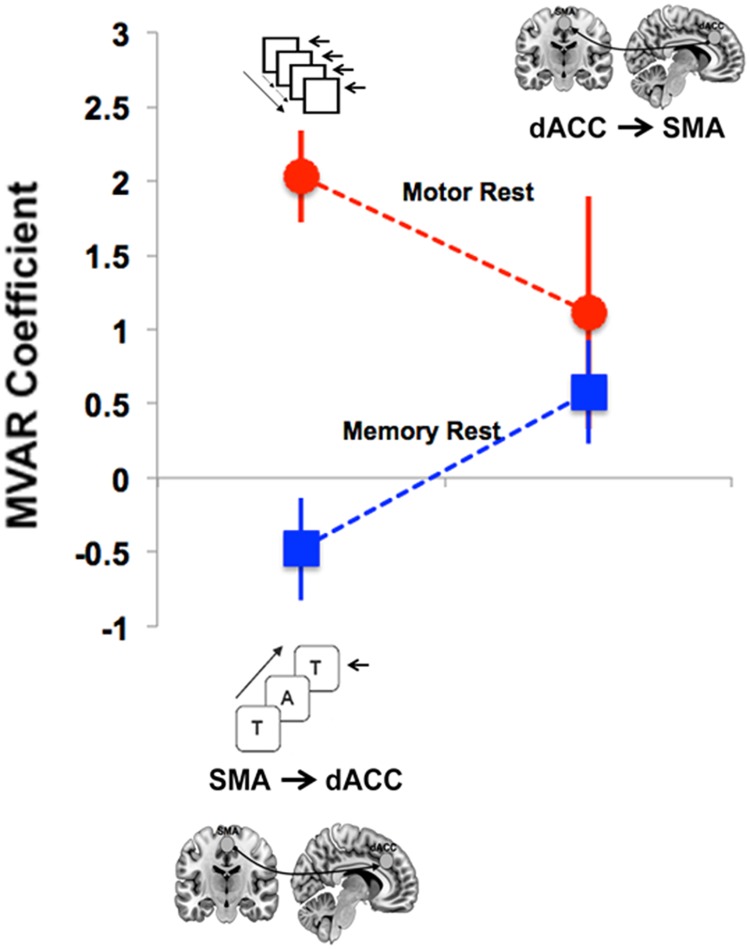
Directed connectivity of resting state signals acquired during each of the Motor (dashed red line) and Working Memory (dashed blue line) tasks showed a significant Within-Task x Direction interaction. The directed FC between the SMA and the dACC was greater during the Motor task than the Memory task with the inverse trend observed for the opposite direction. The greater task-specific directed connectivity of rsfMRI signals from the SMA to the dACC provides compelling evidence for the directional and functional role of resting signals in potentiating the brain for action (see subsequent analyses where increased dACC to SMA signals were observed during motor function). Error bars are ± sem.

The two-way ANOVA following MVAR modeling provided evidence of differences in directional FC in the resting state: A main effect of task (REST [Motor] > REST [Memory]) (*F* = 7.365, p<0.02) was complemented by a significant Task x Direction interaction (*F* = 6.265, p<0.03). Post-hoc tests revealed a significant difference in conditions on the SMA → dACC pathway (*t* = 8.7, *p* < .0001, corrected), indicating that the dFC of this pathway was significantly greater for REST [Motor] than for REST [Memory]. This effect is depicted in Fig 2.

#### Task-related (Motor vs. Memory) dFC of dACC ←→ SMA

**Fig 3:**
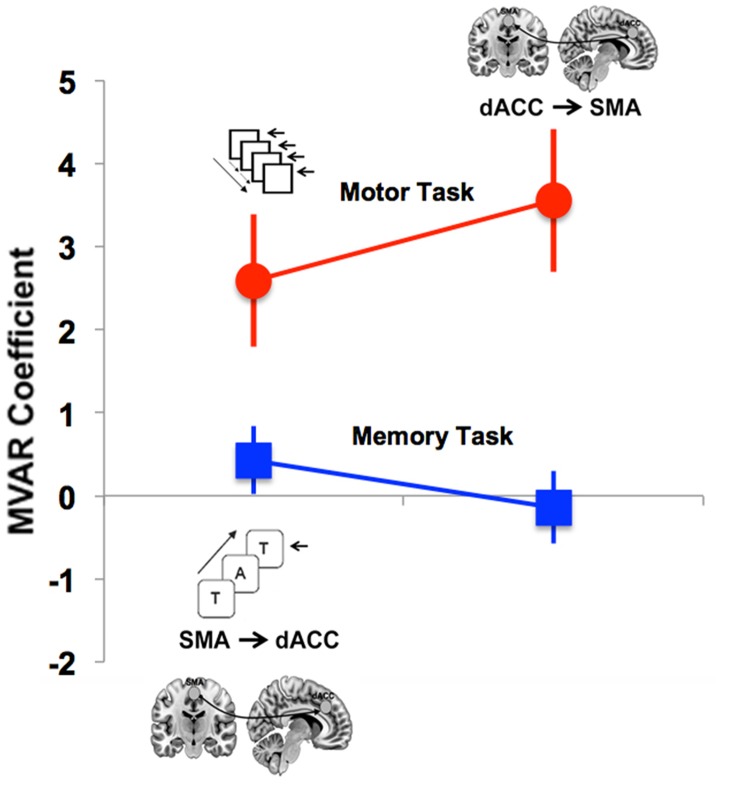
Directed connectivity analyses of task-based data for the visuo-motor (red) and working memory (blue) tasks showed a significant main effect of task (Motor > Memory). Post-hoc tests indicated that the directed connectivity from the dACC → SMA was greater during the Motor than the Memory tasks. Notably, these directional effects appear to complement the corresponding resting analyses (Fig 2). Error bars are ± sem.

The two-way ANOVA following MVAR modeling provided evidence of differences in directional FC between the two Task states: A main effect of Condition (Motor > Memory) (*F* = 18.21, *p* < .01) was observed. Post-hoc tests revealed differences between the dACC → SMA pathway (*t* = 4.65, *p* < .01, corrected) in the two tasks (see Fig 3), specifically elevated FC in the motor task relative to the memory task. By contrast, a significant between-task difference was not observed for the SMA→dACC pathway (*t* = 2.93, *p* > 0.05, corrected).

#### Within-task dFC of dACC ←→ SMA

**Fig 4:**
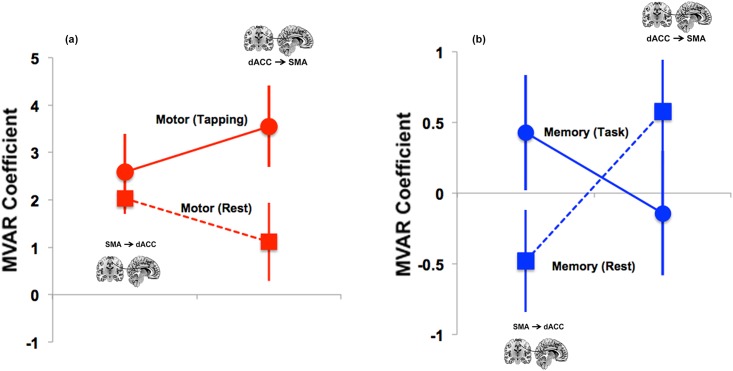
(a) Directed connectivity analyses within the Motor task assessed effects of State (Task vs. Rest) and Direction. As seen, a State x Direction interaction was observed. The dACC → SMA connectivity increased when participants transitioned to the task-active state, relative to the rest state (*t* = 3.73, *p* < .05, corrected). (b) The depicted crossover for the interaction in the Memory condition was not significant (corrected). Error bars are ± sem.

Within each of the Motor and Working Memory tasks, we conducted separate two-way ANOVAs to assess effects of the Task state (Active vs. Rest) and Direction. For the Motor task, we observed a significant Task x Direction interaction (*F* = 9.287, *p* < 0.01) with no other significant effects. This effect is depicted in Fig 4. As seen, during the Motor task, the dFC from dACC → SMA was greater than from SMA → dACC. However, during Rest, the opposite pattern was observed. By comparison, during Memory, a crossover interaction between direction and Condition was observed, though this interaction was not significant (corrected).

Within each of the Motor and Memory tasks, we also assessed the effects of Task Condition (Motor: Periodic vs. Random; Memory: 2-Back vs. 0-Back) and Direction on dFC. Whereas no effects were observed for the Memory-related data, the Motor task was associated with two significant effects: a main effect of Task Condition (*F* = 12.11, *p*<0.007), and a significant interaction (*F* = 5.607, *p*<0.05). Post-hoc analyses revealed that the dACC → SMA connectivity was greater during Periodic relative to the Random epochs (*t* = 4.623, *p*<0.001).

### Connectivity analyses (dACC ←→ M1)

#### Resting-state dFC of dACC ←→ M1

The two-way ANOVA following MVAR modeling provided evidence of a main effect of task (*F* = 8.112, *p*<0.02), but no other significant effects. This main effect resulted from greater connectivity during the Rest blocks of the Motor task than of the Memory task.

#### Task-related (Motor vs. Memory) dFC of dACC ←→ M1

No significant main effects or interactions were observed for these analyses.

#### Within-task dFC of dACC ←→ M1

Across analyses, only the Motor task evinced any significant effects: dFC was greater during Periodic than the Random conditions (*F* = 6.548, p<0.03).

In general, connectivity analyses revealed that dACC **←**→ SMA, rather than the dACC **←**→ M1 interactions, were more substantially modulated by effects of Task, and Rest within Task.

### Additional explorations

**Fig 5:**
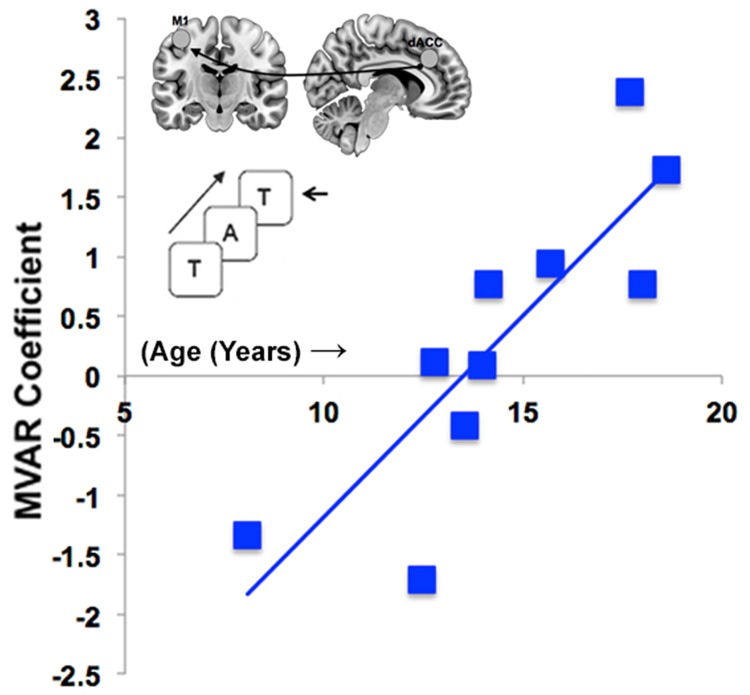
Developmental effects on dACC → M1 connectivity during the Memory task. The figure depicts an increase in dACC → M1 directional connectivity through adolescence during the Memory task. The effect was the only developmental effect of age on any of the connectivity measures. The specificity of this effect implies that age-related immaturity of dACC → M1 interactions may be specific to the more demanding working memory task.

We conducted two additional sets of exploratory analyses to assess the relationships between metrics of dFC and (a) behavioral performance and (b) developmental effects. (a) dFC and behavior: dFC metrics for the bi-directional interactions between the dACC, SMA and M1 during task-based processing were submitted to regression analyses against behavioral performance for each of the Motor conditions (based on response latencies) and Memory (based on sensitivity, *d’*)[20] tasks. Twelve regression analyses were conducted but none of the conducted tests approached significance (*F*s<1.8, *p*s>.2). The null results suggest that in this sample, behavioral proficiency and connectivity estimates were independent, confirming that the relationship between behavior proficiency and fMRI metrics remains variable across published studies [21, 22]. (b) Developmental Effects on Connectivity. We also explored development effects by regressing age on dFC measures of all directions and subnetworks. Sixteen regression analyses were conducted to uncover possible age-related modulation of dFC driven by Task, Task condition or Rest (within Task). Only one regression analysis was significant. During the Memory task, dACC → M1 dFC increased significantly with age (*t* = 4.20, *p* < .05, corrected) (Fig 5).

## Discussion

The goal of our study was to assess dFC between subnetworks within the functional circuitry interconnecting dACC, SMA and M1 (see Fig 1) during both task-active states and resting states that bookended task-active epochs. Understanding of directional effects was of paramount interest; hence Direction was a principal factor evaluated in all the employed analyses of variance. We now reprise our results and discuss plausible mechanistic bases for the observed effects.

First, when considering dFC during rest epochs, SMA → dACC functional connectivity was greater during the Motor than the Memory paradigm, but did not significantly differ in the direction of dACC → SMA (Fig 2). Thus, *the task-context of the rest condition* exerted systematic effects on the direction of the dFC. That is, the fact that the SMA → dACC dFC was significantly stronger in the Motor rest period than the Memory rest period indicates that the two rest periods differed. This task-related dFC difference at rest suggests at least three possibilities to attribute these effects: a) to task-related sets, b) to motor anticipation of the upcoming motor block [23], or c) as an aftermath of motor activity [24]. Our experimental designs do not allow us to disambiguate these possibilities.

The second main finding of this report is that the task-related dFC of dACC → SMA was significantly greater for the Motor than for the Memory paradigm (Fig 3), but did not significantly differ for SMA→dACC. Thus, dFC during task-epochs revealed connectivity patterns in the opposite direction to that of resting signals. This effect was confirmed *within* the Motor paradigm (Fig 4): dACC → SMA functional connectivity was greater during the task-active state than the resting state. Finally, age-related developmental analyses revealed an effect on dFC only during the working memory paradigm: increased age was associated with an increase in dFC of the dACC → M1 pathway (Fig 5). Notably, these effects correspond to the observed behavioral effects, wherein increased age predicted an increase in sensitivity only during the working memory paradigm (see Results section).

Although our methodology does not generally reveal physiological mechanism, these results are highly suggestive of a potential mechanism by which SMA influences dACC at rest. Since the resting directional asymmetry between SMA and dACC is reversed from that during the Motor task, the results suggest that SMA neurons exert an excitatory bias on dACC neurons when the motor system is at rest, and that this bias facilitates the subsequent reverse influence (i.e., dACC → SMA) during the motor coordination task. Such a bias may be related to set-related attention, to motor anticipation, or as an aftermath of motor activity.

### Resting state effects on task-active processing

Is activity in the resting state random or task-related [25]? If random, it is likely that: a) there will be little structure in the RS data, which likely reflect, in large part, random noise that is fed into hemodynamic signals in the absence of task-induced effects; and b) there will be little evidence of systematic relationships between RS FC and task-induced FC within networks. With respect to the first point, some studies have indeed argued that multi-factorial sources of non-neuronal artifact (head motion, arterial CO2 concentration) have to be closely accounted for in assessing RS signals [26–28]. Moreover, correlations between regions may not reflect true cortico-cortical intrinsic connectivity but may reveal synchronization to common thalamic inputs [29] or additional poorly understood confounds [30]. Nonetheless, increasing evidence has unearthed systematic, if not entirely well understood, relationships between RS FC and the structural anatomy of the brain [6, 31], suggesting that RS connectivity has a “non-random” relationship with brain structure. Also, with respect to the second point, even more compelling evidence suggests that intrinsic functional connectivity at rest captures some of the functional characteristics of task-active brain networks [7, 32, 33]. Thus, FC analyses of RS signals have revealed connectivity patterns that broadly mirror the expected organization of task-active functional brain networks [34]. These FC patterns, which lie outside of the default mode network, suggest that resting-state functional connectivity may underpin cognitive network architectures in task-active states. That is, the correlated properties of subnetworks at rest might facilitate engagement of those networks when a task is induced. In summary, previous FC studies give good reason to believe that resting-state activity is in many instances related to the task-active state. The present study is the first to use dFC analysis to support this conclusion. However, we caution that this speculation is incomplete and unclear because the precise role of external inputs in changing the dynamics of the BOLD response is incompletely understood.

Recent investigations using a large-scale field model of neuronal dynamics [35] applied to fMRI BOLD data illustrate this potential complexity. During the fMRI study by those authors, subjects alternated between an extended resting state scan (eyes closed) and an extended visual vigilance task (detecting changes in the luminance of a cross-hair). The principle effect of the vigilance task was found to be a reduction in the variance of the fMRI signal (Fig 7, p 6) with relatively minor changes in undirected functional connectivity between networks. The variance reduction was taken to indicate entropy reduction, and its functional role was interpreted as increasing “… the information capacity of brain networks by enlarging the volume of possible activity configurations at rest and reliably settling into a confined stimulus-driven state to allow better transmission of stimulus-related information [36].” In their interpretation, the “functional connectome” in the resting state generally provides a preparatory state in a flexible large-scale network for rapid and efficient information processing in the cognitive domain in which the task is induced [37]. We note that our applied models of connectivity (dFC) and results (asymmetric dFC on specific subnetworks in the rest and task state) are consistent with this notion and further indicate that this preparatory role of the resting state may be spatially specific.

### Directed and undirected functional connectivity

Previously cited studies have demonstrated a number of large-scale brain networks having statistical functional relationships that are similar in task and rest conditions. Typically, task and rest data in these studies have been acquired in separate scans (but usually in the same scanning session). Undirected functional connectivity (uFC) models of these data make relatively weak assumptions relating to processes but also confer some advantages over dFC measures, such as were used here. For one, uFC measures provide greater *statistical* reliability than dFC measures. Also, they do not require such strong assumptions, nor do they require as precisely modeled time series [10, 38]. However, as we have previously shown, uFC can be less sensitive to detecting task-induced network interactions than dFC [12]. In a case where the *functional hierarchy* of network interactions (e.g., dACC to SMA) [13] is particularly relevant, dFC reveals asymmetric task-induced interactions between hierarchical levels that are sensitive to the precise task characteristics, whereas uFC has not. Moreover, resting-state signals that may be yoked to task context, and that affect task blocks of BOLD activity, in all likelihood possess different *functional* properties than those acquired entirely free of task epochs. However, we caution that, as some uFC analyses have suggested [39], task-specific resting-state FC may not be entirely due to resting-state functional modulation of task periods. Firstly, the deployment of behavioral tasks may induce nonfunctional higher-frequency components in the BOLD signal that are confined to task-specific epochs. In this case, BOLD connectivity in task-specific rest epochs would not have a functional effect on corresponding task-active epochs. Secondly, in oscillating paradigms (even those that do not involve learning or changes in behavioral proficiency over time), rest epochs coincide with periods of psychological recovery during which participants may anticipate the onset of the upcoming task-related epoch. In this case also, BOLD connectivity in task-specific rest epochs would not effect the corresponding task-active function. In each of these examples, it might appear that task-specific rest epochs functionally modulate bookended task epochs, and thus might appear to differ from context-free rest epochs, whereas the true effect would not actually be one of functional modulation.

The single developmental effect within our limited sample is also notable for its close relationship to the observed behavioral data. Whereas behavioral metrics for the motor task were not statistically predicted by chronological age, performance on the working memory task was (see Methods). Chronological age predicted an increase in the sensitivity to detecting targets in the working memory task. In the developmental analyses of dFC, we observed that the only pathway that showed a developmental effect for either of the tasks or conditions, was the dACC → (left) M1 pathway during working memory. In general, dACC involvement in working memory is well-established in health and disease [14, 15, 40], as are developmental changes in proficiency and brain regions associated with this domain [41]. The age-associated increased in dFC between dACC and M1 may reflect increased functional maturation of subnetworks between dACC, directly involved in cognitive and motor control, and M1, the principle cortical motor output region. These results are exploratory, but as with other cross-sectional analyses of working memory-related fMRI signals in development [42], they suggest that functional interactions between cognitive and motor systems are dynamic within tasks, and across development. That is, evidence for functional maturation within the same group of participants may depend on the tasks used to modulate network activity.

There may be a tendency to downplay our results because they are exploratory (given the relatively small sample size and the limited demographic characteristics of the participant sample). Nevertheless, we note that the within-participant’s structure of our experimental design, and the deployment of specifically constrained models of connectivity [10, 11] that are linked to our theoretical focus are advantages. Given the complex (and as yet poorly understood) relationships between structure and function in the brain [43], their poorly understood relevance for cognitive ontologies [44], and the limitations of fMRI recording [45, 46], perhaps all fMRI data and analyses are exploratory and consistent with a process of discovery.

### Limitations and conclusions

The relatively modest sample size in our analyses is a limitation in part driven by the need to include participants with *both* motor and memory data. We have attempted to mitigate the effects of the small sample with conservative statistical approaches for the dFC analyses (see Methods). Nevertheless this aspect may limit the generalizability of the findings. Moreover, given the experimental design, and the limitations on the fMRI signal, it is impossible to ascribe a *precise functional* role to the observed effects particularly for the resting epochs, as there are no task-driven modulators of the fMRI response during those intervals. These limitations notwithstanding, our explorations make a compelling contribution to the literature that seeks to discover functional and possibly specific roles for resting state connectivity in the brain. Our work suggests that, within the dACC ←→ SMA subnetwork, bottom-up dFC (from SMA to dACC) during a resting state that is specific to a motor coordination task may act to potentiate the top-down dFC (from dACC to SMA) exerted during the bookended task. The results indicate that such SMA→dACC potentiation occurs in motor, but not working memory, tasks. This class of analysis, applied to the experimental design employed, makes an incremental contribution to our understanding of the constructive properties of the resting brain.

## Supporting information

S1 File Motor paradigm data: Eigenvariate values based on data extraction from each of the M1, the SMA and the dACC, for each of the study participants are included in “S1 File.xls”. (XLS)

S2 File Working memory paradigm data: Eigenvariate values based on data extraction from each of the M1, the SMA and the dACC, for each of the study participants are included in “S2 File.xls”. (XLS)
